# Traumatic optic neuropathy-associated progressive thinning of the retinal nerve fiber layer and ganglion cell complex: two case reports

**DOI:** 10.1186/s12886-019-1232-9

**Published:** 2019-11-07

**Authors:** Won June Lee, Eun Hee Hong, Hae Min Park, Han Woong Lim

**Affiliations:** 1 Department of Ophthalmology, Hanyang University Hospital, Hanyang University College of Medicine, 222-1, Wangsimni-ro, Seongdong-gu, Seoul, 04763 South Korea; 2 Department of Ophthalmology, Hanyang University Seoul Hospital, Seoul, South Korea; 3 Department of Ophthalmology, Hanyang University Guri Hospital, Guri, South Korea

**Keywords:** Traumatic optic neuropathy, Optical coherence tomography, Neuro-ophthalmology

## Abstract

**Background:**

Traumatic optic neuropathy (TON) is a form of optic nerve injury that occurs secondary to trauma and is etiologically associated with acute axonal loss with severe vision loss. Here, we reported longitudinal changes in the peripapillary retinal nerve fiber layer (RNFL) and macular ganglion cell complex (GCC) using wide-field swept source optical coherence tomography (SS-OCT) in two cases of TON and identified the source of the damage.

**Case presentation:**

(Case 1) A 65-year-old man was admitted to the hospital due to an injury in the right eye (OD) and was subsequently diagnosed with indirect TON. He was then treated with high-doses of intravenous steroids. Wide-field SS-OCT was performed at the baseline and after 1 day, 2 days, 1 week, 1 month, and 4 months. The wide-field deviation map detected thinning earlier in the macular GCC than in the peripapillary RNFL. (Case 2) A 63-year-old man was admitted to the hospital with a fractured left maxilla-zygomatic complex attributed to blunt-force trauma to the head and loss of vision in his left eye (OS). He was diagnosed with indirect TON and treated with high-doses of intravenous steroids. Wide-field SS-OCT was performed at the baseline and after 1 week, 2 weeks, 2 months 5 months, and 7 months. The wide-field deviation map detected thinning earlier in the peripapillary RNFL than in the macular GCC.

**Conclusions:**

Wide-field SS-OCT facilitated the identification of various sequential progression patterns in patients with TON. Furthermore, the area in which the structural damage was first detected was seen differently in the peripapillary and macular deviation maps for each case. Thus, wide-field imaging, which includes the macular and peripapillary areas, are useful in monitoring TON.

## Background

Traumatic optic neuropathy (TON) is a type of optic nerve injury that occurs secondary to trauma and has been etiologically associated with acute axonal loss with severe vision loss [1]. Indirect TON refers to a variation of TON that is caused by forces transmitted at a distance from the optic nerve after blunt force trauma to the head. This type of optic nerve injury is typically observed in the optic canal [2]. Spectral-domain optical coherence tomography (SD-OCT) has been widely used to measure structural changes in the retinal layers, and this technology was used to monitor several retinal diseases and optic neuropathies, including TON [3–5].

Recently, a number of reports have suggested the integration of SD-OCT analyses of the peripapillary and macular areas using an embedded software (PanoMap) or a simultaneous interpretation of both areas to diagnose or determine disease progression in glaucoma [6–8]. Changes in these two areas have also been reported in other non-traumatic optic neuropathies [9, 10]. Advancements in technology, such as swept-source OCT (SS-OCT), have facilitated the use of wide-field to cover both the peripapillary and macular areas [6, 11, 12].

A number of studies have clinically demonstrated morphological changes in the thickness of the retinal layer using OCT or scanning laser polarimetry in patients with TON [13–17]. However, only one study has directly compared the thickness of the peripapillary retinal nerve fiber layer (RNFL) with that of the macular ganglion cell complex (GCC) after TON [13]; additionally, to the best of our knowledge, wide-field scanning with SS-OCT in TON has not been reported yet. Comparing the two areas may provide information about the progression and pathophysiology of the disease. Here, we reported longitudinal changes in the peripapillary RNFL and macular GCC using wide-field SS-OCT in patients with TON and identified the area where the damage was first detected.

## Case presentation

### (case 1)

A 65-year-old man was admitted to the hospital after he presented with symptoms such as periorbital swelling and bruising in the right eye (OD) caused by blunt force trauma to the head. Despite his injury, he could adequately perform the finger counting test and demonstrated an intraocular pressure (IOP) of 17 mmHg in OD. The slit lamp and fundus examination was normal. His OD demonstrated mid-dilated pupil, along with relative afferent pupillary defect. Computed tomography did not reveal any significant abnormalities, such as bone fractures, except for swelling in the periorbital soft tissue. Following the diagnosis of indirect TON, the patient was immediately treated with high-dose intravenous steroids (3000 mg of intravenous methylprednisolone in total).

**Fig. 1:**
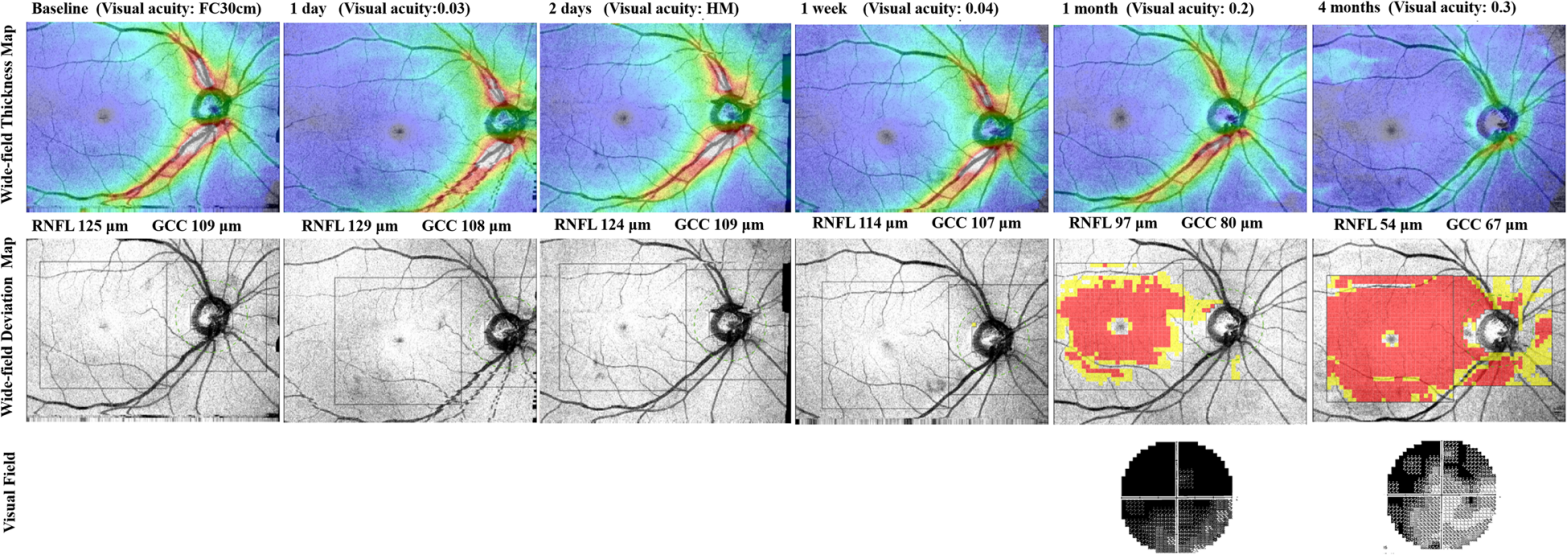
Serial wide-field optical coherence tomography angiography (OCT) of the right eye of 65-year-old man after injury. Identification of progressive thinning in both the peripapillary retinal nerve fiber layer (RNFL) and macular ganglion cell complex (GCC) using a wide-field thickness map. The wide-field deviation map shows that thinning is first detected in the macular GCC than in the peripapillary RNFL. Changes in color (yellow and red) in the peripapillary RNFL at the 4-month time-point indicate RNFL thinning. The area marked red in the macular GCC at the 1-month time-point indicates GCC thinning

Wide-field SS-OCT was performed at baseline and after 1 day, 2 days, 1 week, 1 month, and 4 months. During the follow-up periods, his IOPs were within the normal range and no further complications occurred. The wide-field thickness map revealed a gradual thinning of the peripapillary RNFL and macular GCC. The wide-field deviation map showed that thinning was detected first in the macular GCC than in the peripapillary RNFL (Fig. 1).

### (case 2)

A 63-year-old man was admitted to the hospital with fractured left maxilla-zygomatic complex caused by blunt force trauma to the head. He complained about loss of vision in his left eye (OS), and his visual acuity permitted him to identify hand movements. The IOP in his OS was 20 mmHg. The slit lamp and fundus examination was normal. The pupil was normal sized, and relative afferent pupillary defect was observed in OS. Computed tomography showed normal optic nerve and without any of the following features: displaced fracture fragments compressing the optic nerve, hematoma, bleeding in the ethmoid sinus spaces, cerebral injury, and bone fracture in the optic canal. Following the diagnosis of indirect TON, the patient was immediately treated with high-doses of intravenous steroids (3000 mg of intravenous methylprednisolone in total).

**Fig. 2:**
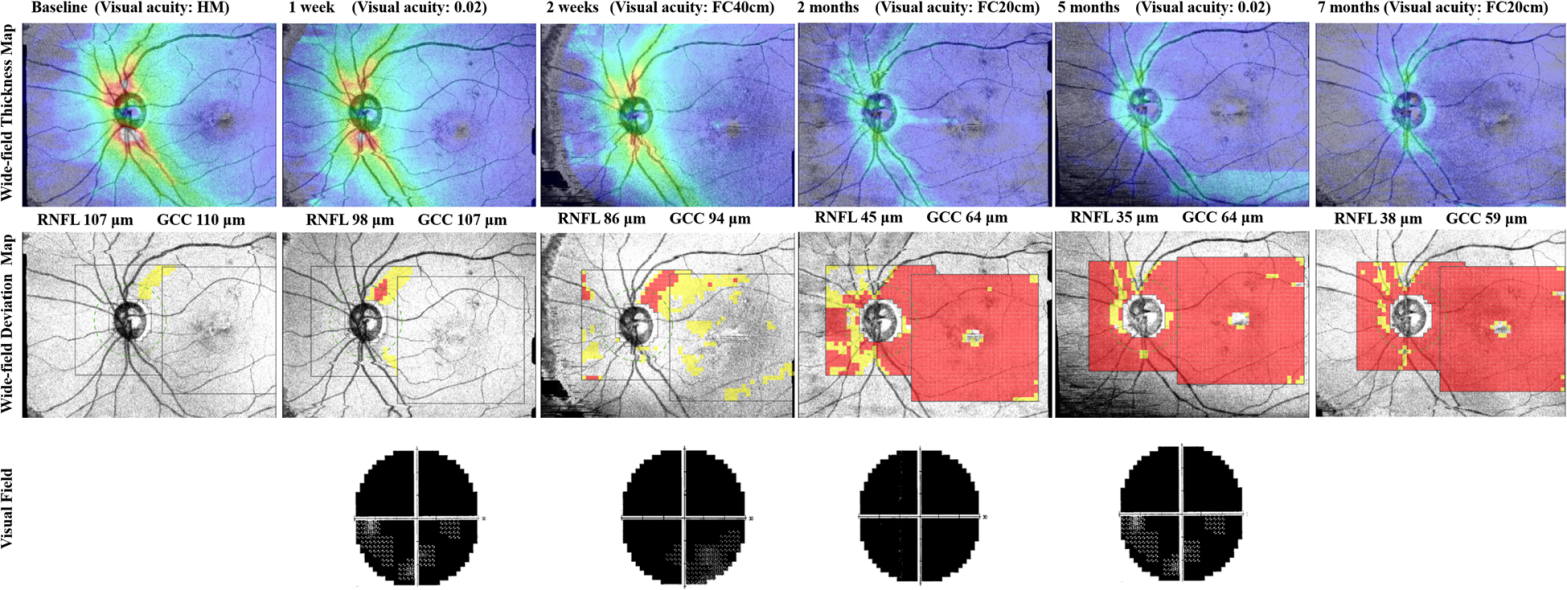
Serial wide-field optical coherence tomography angiography (OCT) of the left eye of 63-year-old man after injury. Detection of progressive thinning in both the peripapillary retinal nerve fiber layer (RNFL) and macular ganglion cell complex (GCC) using a wide-field thickness map. The wide-field deviation map shows that thinning is first detected in the peripapillary RNFL, followed by in the macular GCC. Color changes (yellow and red) in the peripapillary RNFL from baseline to 7 months indicate progressive RNFL thinning. The area marked red in the macular GCC after 2 months indicates GCC thinning

Wide-field SS-OCT was taken at baseline and after 1 week, 2 weeks, 2 months, 5 months, and 7 months. During the follow-up periods, IOPs were in the normal range, and no further complications occurred. The wide-field thickness map revealed that the peripapillary RNFL and macular GCC were thinning gradually. The wide-field deviation map showed that thinning was detected first in the peripapillary RNFL than in the macular GCC (Fig. 2).

## Discussion and conclusions

In this case report, we presented sequential structural changes associated with TON in both the peripapillary and macular areas over a wide-field area using SS-OCT and identified the area where the damage was detected first. The peripapillary and macular deviation maps revealed differences in the area at which the structural damage was first detected in the two cases. To the best of our knowledge, this is the first study to report the sequential relationship with regard to the areas in TON.

A number of reports have suggested that retinal thinning is detected in TON both at the peripapillary and macular areas [13–17]. However, these studies did not provide sufficient information regarding the area that first demonstrated the damage in TON. One case report that directly compared the thickness of the peripapillary RNFL and GCC after TON demonstrated that the time course of the reduction in the macular area was similar to that observed in the peripapillary RNFL. The authors of the same study suggested that the loss of retinal ganglion cells and related axons continued at rates similar to those of the loss of axonal injury [13].

In the first case, the wide-field deviation map revealed that structural damage was first detected in the macular GCC than in the peripapillary RNFL. Unlike the name of the disease (TON), which indicates that the main lesion of the disease is concentrated in the optic nerve, retinal thinning may first be detected in the macular area. Alternatively, in the second case, wide-field deviation map showed that thinning was detected earlier in the peripapillary RNFL than in the macular GCC. In both cases, the wide-field thickness map showed gradual thinning of the retinal layer in both the peripapillary and macular areas. However, wide-field deviation map, which compared the measured thickness to the embedded normative database, demonstrated that the location where damage is first shown differed depending on the cases.

Considering diseases such as glaucoma where it is imperative to have an accurate judgment of disease progression, several reports have recently investigated the initial point from where the progression of the disease originates and is subsequently detected [8, 18, 19]. Despite several controversial evidences, Kim et al. reported that early glaucomatous structural damage can be observed earlier in the macular ganglion cell-inner plexiform layer (GCIPL) than in the peripapillary RNFL with SD-OCT. [20, 21] TON is commonly caused by indirect injury to the optic nerve, which is thought to be the result of a shock that has been transmitted from an orbital impact to the intracanalicular portion of the optic nerve [1]. Although the initial location of the lesion may be different, both diseases are similar to an extent due to the fact that an axonal injury, and not the retinal ganglion cell, may be the point of origin. Therefore, the structural damage associated with TON that was observed in case 1 may also be detected first in the macula.

The deviation map visualizes the thinned area comparing with the averaging normative database of the control group, suggesting that the progression of damage over time may differ depending on the baseline anatomical variation in individual patients. In case 2, the structural damage of TON was detected first in the peripapillary area.

Recently, one group reported that functional damage in TON was well correlated with the macular GCIPL status [14]. In advanced glaucoma, evaluation of the peripapillary RNFL was less clinically useful due to a “floor effect” of the thickness of the RNFL With advanced loss, RNFL thickness never falls below 40 μm due to the assumed presence of residual non-neuronal tissues [22]. At this stage of the disease, evaluation of the macular GCIPL using SD-OCT was considered to be a more efficient tool to judge progression [23]. Similarly in TON, OCT images of the macular area could be more useful than those of the peripapillary area to evaluate the functional aspects of the disease.

Wide-field OCT can simultaneously visualize the thickness of the neural tissue in both the peripapillary and macular areas. Therefore, wide-field OCT can be used to specifically identify TON-associated nerve damage and also to obtain information about the progression of TON in a relatively wide area. Unfortunately, relationship between the structural and functional impairments could not be elucidated with only two cases and may be understood in a future study with a larger sample size.

To conclude, in this case report, wide-field SS-OCT allowed us to confirm various sequential progression patterns in patients with TON, along with wide-field imaging of the macula and peripapillary areas, which are useful to monitor TON.

## Data Availability

The datasets used and/or analyzed during the current study are available from the corresponding author on reasonable request.

## Abbreviations

GCC: Ganglion cell complex
GCIPL: Ganglion cell - inner plexiform layer
OD: right eye
OS: left eye
RNFL: Retinal nerve fiber layer
SD-OCT: Spectral-domain optical coherence tomography
SS-OCT: Swept-source optical coherence tomography
TON: Traumatic optic neuropathy
